# “Adherent” versus Other Isolation Strategies for Expanding Purified, Potent, and Activated Human NK Cells for Cancer Immunotherapy

**DOI:** 10.1155/2015/869547

**Published:** 2015-06-16

**Authors:** Senthamil R. Selvan, John P. Dowling

**Affiliations:** ^1^Division of Solid Tumor, Department of Medical Oncology, Thomas Jefferson University, Curtis Building, Suite 1024B, 1015 Walnut Street, Philadelphia, PA 19107, USA; ^2^Immunology and Microbial Pathogenesis Graduate Program, Thomas Jefferson University, Philadelphia, PA 19107, USA

## Abstract

Natural killer (NK) cells have long been hypothesized to play a central role in the development of new immunotherapies to combat a variety of cancers due to their intrinsic ability to lyse tumor cells. For the past several decades, various isolation and expansion methods have been developed to harness the full antitumor potential of NK cells. These protocols have varied greatly between laboratories and several have been optimized for large-scale clinical use despite associated complexity and high cost. Here, we present a simple method of “adherent” enrichment and expansion of NK cells, developed using both healthy donors' and cancer patients' peripheral blood mononuclear cells (PBMCs), and compare its effectiveness with various published protocols to highlight the pros and cons of their use in adoptive cell therapy. By building upon the concepts and data presented, future research can be adapted to provide simple, cost-effective, reproducible, and translatable procedures for personalized treatment with NK cells.

## 1. Introduction

Natural killer (NK) cells are innate immune cells that comprise 5–20% of peripheral blood mononuclear cells (PBMCs) [1]. As their name suggests, NK cells have an intrinsic ability to spontaneously lyse virally infected and cancerous cells, a function that is normally mediated by a balance of activating receptors (e.g., NKG2D) and inhibitory receptors (killer immunoglobulin-like receptors (KIR), NKG2A) [2]. The activation signals are triggered by receptors like NKG2D, which recognize stress ligands like MICA and MICB on potential target cells, and CD16, which binds to the Fc portion of IgG antibodies to initiate antibody-dependent cellular cytotoxicity (ADCC) of a target cell. Conversely, inhibitory signals triggered by KIR are capable of thwarting this activation when bound to self-MHC molecules on the target cell [3]. This prevents NK cells from lysing the body's own cells and allows effective targeting of virally infected or tumor cells, which commonly downregulate MHC as an immune escape mechanism [4]. There are two subsets of NK cells in the blood based on phenotype and function. They are CD56^bright^CD16^dim⁡^, which tend to play an immunoregulatory role releasing cytokines like IFN-*γ*, TNF-*β*, and GM-CSF [5], and CD56^dim⁡^CD16^bright^, which are more cytotoxic and express higher levels of CD16 [6]. When activated, these NK cells alter their receptor expression and produce higher amounts of perforin and granzyme as well as upregulating receptors involved with dendritic cell crosstalk [7].

NK cells are derived from the common lymphoid progenitor, along with T and B cells [8]. They are traditionally considered innate immune cells since they utilize germline-encoded receptors, mentioned above, to elicit their effector functions rather than undergo genetic rearrangement of immensely diverse antigen receptors. Emerging evidence, however, suggests that NK cells are more like the adaptive immune system than previously thought, which could provide even more advantages for their use in immunotherapy [9]. They are capable of undergoing a selection process to prevent autoreactivity [10], survive for long periods of time* in vivo* [11], and undergo robust memory-like responses upon a secondary challenge with antigen [12–14].

The antitumor effects of NK cells have long been realized in* in vitro* and* in vivo *studies [15]. More recent* in vivo *studies have further elucidated NK cells' role in tumor immunity, showing that, upon depletion of NK cells using NK1.1 antibody, mice had impaired rejection of tumor cells and enhanced tumor metastasis [16]. In addition, the presence of NK cell receptor ligands and lack of MHC Class I expression on target cells have been shown to play a role in tumor rejection [17, 18]. Importantly, this NK cell-mediated tumor rejection can also induce the development of tumor-specific T cells, capable of rejecting a secondary tumor challenge [19]. However, several immunosuppressive events can occur during tumor development that can inhibit NK cell cytotoxic function and affect tumor rejection. For example, tumor-associated NK cells are shown to downregulate activating receptors and become less cytotoxic [20, 21] and downregulation of essential chemokines like CCL2 can prevent NK cell trafficking to the site of a developing tumor [22, 23]. There is also evidence that suggests that some genetic mutations that affect NK cell activation result in an increased risk to develop cancer [24].

Due to NK cells intrinsic antitumor activity, they have long been hypothesized to play a central role in new cancer immunotherapies [25]. Administration of tumor-specific antibodies like rituximab (anti-CD20) and trastuzumab (anti-Her2/neu) is thought to rely on NK cell-mediated ADCC for their antitumor activity [3, 26]. Further approaches are attempting to specifically boost NK cell ADCC, like the use of bispecific killer cell engagers (BiKEs), which initiate CD16-mediated tumor-specific lysis [27, 28]. The main focus of NK cell immunotherapy has been developing protocols to isolate NK cells from patients' peripheral blood, expand activated NK cells to large numbers, and return them to the patient in the hope of a potent antitumor effect [29–31]. Multiple approaches have been investigated to develop NK cell adoptive cell therapy using various isolation and expansion methods. For all protocols, patient's PBMCs must first be isolated through leukapheresis, a density gradient using Ficoll-Paque solution, or another Good Manufacturing Practice- (GMP-) compliant method. Further NK cell isolation can be achieved using magnetic cell sorting (MACS) to separate CD3^−^CD56^+^ cells from PBMCs [32] or T cell depletion using anti-CD3 antibody (OKT-3) [33]. Expansion and activation of NK cells has been achieved using various cytokines, namely, IL-2 [34], or using “feeder cells” that selectively activate NK cells through cell-cell contact [35, 36]. Once expanded, these NK cells have been tested to treat a wide variety of cancers, including both liquid and solid tumors [37, 38]. However, the development of GMP-compliant procedures that ensure consistent isolation and expansion of NK cells that retain their potency as well as the safety of the final product for the patient remains a major hurdle [21].

In the present study, we compare a simple and cost-effective method of “adherent” enrichment and expansion of NK cells, aimed at being directly translatable to the clinic, developed in our laboratory with various published NK cell enrichment and expansion methods by considering NK cell purity, potency, expansion, experimental procedure, cost, and the ease of translation to the clinic. These protocols have been divided into four categories based on their expansion methods: no or brief* in vitro* stimulation, cytokines, feeder cells, and, lastly, our “adherent” enrichment and expansion of NK cells.

## 2. No or Brief* In Vitro* Stimulation

Since it is difficult to isolate a large number of NK cells from the peripheral blood, studies have investigated the direct injection of freshly isolated or overnight stimulated NK cells. Miller et al. stimulated MACS CD3-depleted PBMCs overnight in IL-2 supplemented media [39]. This product was generated from PBMCs of haploidentical donors and contained an average of 40% NK cells. Forty-three patients were tested. Five out of nineteen AML patients, that received more intense preconditioning with cyclophosphamide and fludarabine, achieved a complete remission and survival of infused NK cells. To show survival/expansion of the NK cells, the authors used RT-PCR. They also removed expanded NK cells after 14 days during the more intense preconditioning and showed they were capable of lysing K-562 cells.

Rubnitz et al. investigated the use of haploidentical NK cells to prevent relapse of AML patients in first complete remission. Patients were preconditioned with cyclophosphamide and fludarabine followed by infusion of KIR-HLA mismatched NK cells and 6-day IL-2 administration. Engraftment was safe and successful and all ten patients remained in complete remission after two years [40]. Curti et al. treated thirteen AML patients with MACS-purified CD56^+^ NK cells from KIR-HLA mismatched donors that were not stimulated* in vitro* [41]. These authors also preconditioned the patient with cyclophosphamide and fludarabine followed by infusion of 2.74 × 10^6^ cells/kg (product contained both NK and NK-T cells) and IL-2 dose administration. One out of five patients with active disease and two patients in molecular relapse achieved a transient complete response. Three of six patients that were in a complete remission before receiving NK cells were still in remission at the time this work was published. This treatment was also considered safe and feasible.

Stern et al. performed a two-center phase II trial treating sixteen patients with infusions of purified NK cells after a haploidentical stem cell transplant [42]. NK cells were isolated using a two-step CliniMACS procedure that depleted CD3^+^ cells and then positively selected CD56^+^ cells. This product was cryopreserved until its use. Four of sixteen patients were alive and still in remission at the time this work was published. However, this result is similar to historical controls and therefore, the NK cells had no apparent effect on relapse. As described, most of these studies involve patients who were in remission from hematopoietic cancers and used some preconditioning or stem cell transplant along with exogenous IL-2. In addition, they used healthy donor derived NK cells that may be more potent antitumor effector cells compared to patient's NK cells. However, this approach is unable to generate large numbers of these NK cells due to the low percentage of NK cells in the peripheral blood especially in cancer patients [21].

## 3. Cytokines

### 3.1. IL-2

The antitumor effects of IL-2 and IL-2 treated lymphocytes have been extensively studied. Particularly, lymphokine-activated killer (LAK) cells have been generated* in vitro* by treating human PBMCs with 200 U/mL IL-2 for several days. This LAK population is highly cytotoxic against tumor cells and has been investigated in preclinical mouse models and clinical trials since the 1980s [43]. Further study of LAK cells showed that although NK cells (CD3^−^CD56^+^) comprised only a small percentage of the population, they were the most potent cytotoxic subset [44]. The ability of IL-2 to expand NK cells and enhance their cytotoxic function has also been studied since the 1980s [45–47]; however, IL-2 will also stimulate T cells. In particular, T regulatory cells (T_regs_) express high levels of the IL-2 receptor (CD25) and can limit the availability of IL-2 to NK cells [48]. For this reason, MACS purification or anti-CD3 depleting antibody is typically used to enhance NK cell purity. This has been effective at expanding NK cells but can cause IL-2 dependence of NK cells as well as toxicity in patients when the cytokine is given at high doses [49].

In 2001, Carlens et al. achieved an NK expansion with relatively high purity using IL-2 and OKT-3 (anti-CD3 antibody) [34]. OKT-3 has been shown to deplete T cells by binding to and internalizing CD3 [50]. By using IL-2 supplemented Cellgro SCGM media with 5% human serum and OKT-3, a 193-fold expansion of 55% pure NK cells was observed after 21 days, as determined by CD3^−^CD56^+^ cells. This initial proof-of-principle study performed in healthy donors was further verified using PBMCs from multiple myeloma (MM) patients. This study, published by the same group, now demonstrated a 1,625-fold expansion of patient-derived NK cells with 65% purity [30]. The expanded NK cells showed increased cytotoxicity against MM target cells and higher percentage of these NK cells underwent degranulation, as determined by CD107a staining. Importantly, these cells were not cytotoxic against normal host cells. This approach also uses all GMP-compliant components. In the initial clinical trial investigating this method, Barkholt et al. expanded CD56^+^ cells (NK and NK-T cells) from PBMCs of haploidentical donors as described above [51]. Each patient in the trial had received an allogeneic stem cell transplant. After a 20-day expansion, CD3^−^CD56^+^ cells expanded an average of 6,000-fold and made up ~35% of the cell product. These cells were infused to five patients with various cancers, followed by two further infusions of cryopreserved cells. Only one patient, who had hepatocellular carcinoma, had a detectable response determined by decreased serum *α*-fetoprotein levels. This approach was also deemed safe albeit with less percent of NK cells. More recently, a large-scale protocol has been adapted to use closed-system bioreactors [52]. By automating this process in a closed system, the ability to undergo clinical trials may now be more feasible.

Expanding NK cells with IL-2 alone would be ideal for the translation of NK cell expansion protocols because GMP-compliant recombinant human IL-2 is widely available and relatively inexpensive. However, the nonspecific properties of IL-2 also cause expansion of contaminating T cells. If expanded PBMCs are from a donor rather than the patient being treated, stringent isolation methods are required to ensure a purified NK cell product to prevent contaminating T cells from causing GVHD in the recipient. These isolation methods can further complicate GMP-compliant protocols, particularly when performing large-scale expansions.

### 3.2. IL-15

Due to the difficulty in achieving large numbers of pure NK cells using only IL-2, other cytokines like IL-7 [53], IL-21 [29], and IFN-*γ* [33] have been investigated to improve NK expansion and potency. Namely, IL-15 has been a major cytokine used to upregulate NK cell activating receptors and induce proliferation [54]. Studies suggest that IL-15 may not help with proliferation but may preserve the viability of NK cells, achieving a better yield [55]. However, more recent studies suggest IL-15 is not required for survival of human NK cells and does not augment or support *T*_regs_ *in vivo* [56]. We also observed similar enhanced viability of NK cells with higher percent lysis of K-562 cells when combining IL-2 and IL-15 in our method of “adherent” enrichment and expansion of NK cells as described below (data not shown; see Section 5).

Klingemann and Martinson showed successful expansion after MACS positive selection of CD56^+^ cells. The use of IL-2 or a combination of IL-2 and IL-15 was compared over a 14-day period under GMP conditions [32]. Although similar expansions occurred using both culture conditions, the combination of cytokines allowed NK cells to lyse a higher percentage of target cells at a 1 : 1 E : T ratio. Starting with a population of 60% CD3^−^CD56^+^ and 30% CD3^+^CD56^+^ after MACS selection, the authors note that this expansion varied greatly and CD3^+^CD56^+^ cells (NK-T cells) expanded 2-3 times as much as CD3^−^CD56^+^ cells (NK cells) although exact percentages were not shown.

Suck et al. published a comparison of IL-2 and IL-15 supplemented CellGro SCGM using GMP-compliant conditions to expand NK cells within a LAK population [57]. OKT-3 was included in the media throughout the first five days to deplete the T cells. Data showed superior long-term NK cell expansion and cytotoxic ability using IL-15 compared to IL-2. Over a 4-week period, an average of 2,320-fold NK cell expansion was observed compared to 1,084-fold using IL-2. The purity of NK cells was 40%–50% after a 15-day culture. In addition, both activating and inhibitory receptors were upregulated in the IL-15 expanded NK cells but not the IL-2 expanded NK cells. This expansion was also modified to use Baxter Lifecell culture bags, showing the reproducibility of this protocol in a closed-system.

While some success has been documented in expanding NK cells with cytokines other than IL-2, a consensus cytokine or method has not emerged to demonstrate a clear advantage. In some cases, combination with IL-2 is needed and when the expansion is compared with IL-2 alone, only a slight benefit is observed. In addition, it would be difficult to test these protocols in clinical trials. Most cytokines, like GMP-compliant IL-15, are much more expensive and less common than IL-2, whereas other cytokines, like IL-21, shown to increase cytotoxicity and cytokine production in NK cells, are not yet available for clinical use [58].

## 4. Feeder Cells

### 4.1. Tumor Cell Lines as Feeder Cells

For more than 20 years, feeder cells have also been considered a potential strategy to expand and activate NK cells [59]. By using cells capable of being lysed by NK cells, like K-562, or those capable of providing crosstalk, like PBMCs [60], activation signaling can be provided to NK cells to stimulate growth and cytotoxic activity. These feeder cells are normally irradiated before coculture to inhibit growth. Genetic modification has been used in many cases to express NK cell activation signals, like 4-1BBL and membrane bound IL-15 (mbIL-15). Most protocols use these feeder cells in addition to IL-2 to achieve dramatic NK expansions.

In 2002, Harada et al. successfully used a Wilms tumor cell line, HFWT, as feeder cells for natural killer cell expansion [35]. HWFT is an NK-sensitive, adherent cell line with downregulated MHC class I expression. On day 0, 1 × 10^6^ healthy donor PBMCs were seeded at a 10 : 1 E : T with HFWT target cells in IL-2 supplemented RHAM*α* media. This method obtained a 401-fold expansion with 77% purity of CD3^−^CD56^+^ cells over a 21-day procedure. This approach also produced better expansion and purity than unmodified K-562 cells. These expanded cells were cytotoxic against live HFWT cells as well as a gliosarcoma cell line. NK cells could also be expanded from a patient and were shown to be cytotoxic against autologous brain tumor cells. Also, a final experiment confirmed the need for cell-cell contact between feeder cells and NK cells to initiate activation. This was performed using cell inserts to prevent direct interaction of feeder and PBMCs in the same well. Only 5% of cells above the filter were NK cells compared to 78% NK cells in contact with the feeder cells, showing that direct cell contact is necessary for activation. This protocol was tested in a clinical trial by the same group in Japan to treat grade-3 and grade-4 glioma patients [61]. Autologous NK cells were isolated from patients with an average purity of 82.2%. In addition, adjuvant low-dose IFN-*β* was administered weekly. Three partial responses and two mixed responses occurred. In a recent follow-up article, the authors mention that this remains a potentially effective therapy but they are investigating vaccine strategies, in part due to the high costs of* ex vivo* expansion [62].

In 2005, Imai et al. genetically modified K-562 cells to express mbIL-15 and 4-1BBL using retroviral transduction [63]. The authors mention several reasons for this approach: NK cells have previously been stimulated with K-562 cells, modified K-562 cells have successfully expanded CD8^+^ T cells, and IL-15 is more potent when presented to NK cells in a membrane bound form [64]. Optimal NK cell number and purity were obtained when culturing with K-562 cells that expressed both IL-15 and 4-1BBL in IL-2 supplemented RPMI-1640 media. Additionally, the transduction of an anti-CD19 chimeric antigen receptor gave NK cells the ability to overcome tolerance of B cell leukemia. Ultimately, NK cells expanded to >1,000-fold after a 21-day protocol. The resulting NK cells were highly cytotoxic against patient leukemia cells. In later work, this method was directly compared to IL-2, IL-15, and IL-21 in a 7-day treatment of PBMCs [29]. Here, NK cells expanded with feeder cells yielded larger number of cells and exhibited higher cytotoxicity against target cells. Next, a large-scale expansion protocol was optimized expanding NK cells with K-562-mbIL15-4-1BBL feeder cells in VueLife bags using IL-2 supplemented Cellgro SCGM media [29]. This approach, named the Natural Killer Cell Activation and Expansion System (NKAES), was geared towards treating acute myeloid leukemia (AML) and expanded cells were shown to lyse tumors cells both* in vitro* and* in vivo*. Also, the authors established a master cell bank using GMP guidelines that included safety measures to ensure no cell growth and no viability of K-562 cells after NK expansion. After seven days, the NK cells had expanded between 33- and 141-fold with 83.1% purity. Efficacy of these expanded cells to treat solid tumors like Ewing sarcoma, rhabdomyosarcoma, neuroblastoma, and osteosarcoma was shown by demonstrating NK cell lysis of cell lines both* in vitro* and* in vivo* [37]. This extensively studied expansion method supports the potential to treat both solid and liquid tumors as well as expand genetically modified NK cells for targeting specific cancers [65].

Several other feeder cell protocols have also been successful expanding NK cells. Denman et al. used irradiated K-562 cells expressing membrane bound IL-21 and IL-2 supplemented media to achieve a mean 47,967-fold expansion of NK cells in 21 days [66]. This can be performed starting with PBMCs or purified NK cells in tissue culture flasks. More recently, this expansion method was shown to generate expanded NK cells from PBMCs from children with neuroblastoma [67]. These expanded NK cells were 83% CD3^−^CD56^+^ cells and were cytotoxic against neuroblastoma cell lines* in vitro* and* in vivo*. Cytotoxicity was increased with the addition of an anti-GD2 antibody. Also, these cells could be cryopreserved and maintain their antitumor activities.

Dowell et al. introduced an ovarian carcinoma cell line (OVCAR) modified to express 4-1BBL and IL-12 using an adenoviral vector [68]. These feeder cells were cocultured with nonadherent PBMCs in IL-2 supplemented media to achieve a 29.7-fold expansion that yielded 70–90% CD3^−^CD56^+^ cells in 7 days and this approach supported 21-day NK cell culture. A phenotypic analysis of the expanded NK cells showed they were mainly CD56^bright^CD16^−^ NK cells and lysed K-562 and OVCAR cell lines.

Berg et al. demonstrated an NK expansion method using an Epstein-Barr virus-transformed lymphoblastoid cell line (EBV-LCL) feeder cells, a cell line shown to be safe for clinical trials [31]. The protocol begins with 2 × 10^8^ NK cells purified using the CliniMACS system, selecting CD3^−^CD56^+^ PBMCs. It requires only one round of stimulation with EBV-LCL feeder cells and continued culture with IL-2 supplemented* X-VIVO* 20 media in Baxter Lifecell bags. This results in up to a 850-fold expansion of 98% pure CD3^−^CD56^+^ cells after a 2-3-week culture [31, 55]. These expanded NK cells will be tested in combination with bortezomib, an FDA approved proteasome inhibitor that has been shown to sensitize tumor cells to TRAIL-dependent NK cell lysis. One potential roadblock could be obtaining the initial 2 × 10^8^ NK cells required to begin this procedure due to the low percentage of NK cells in PBMCs, especially in cancer patients. Also, the expanded NK cells were shown to have IL-2 dependence. When IL-2 was removed from the media or cells were frozen, NK cells became inactivated. Although restoration of the activated NK cell phenotype could be restored by readministering IL-2, this might require administration of IL-2 to the patient. Finally, the authors note that IL-2 dependence could potentially prevent attempts to freeze cells to use for multiple administrations. Preliminary results of a phase I dose escalation study treating chromic lymphoid leukemia (CLL) and other metastatic tumors indicate that administering 1 × 10^8^ NK cells/kg is safe and tolerated. At the time of this report, seven out of fourteen patients had stable disease, two patients had a 30% decrease in serum tumor markers, and one minor response of a metastatic kidney cancer patient was noticed [69].

### 4.2. PBMCs as Feeder Cells

Another strategy shown to expand NK cells is the use of irradiated PBMCs as feeder cells. In 2002, Luhm et al. published a GMP-compliant NK cell expansion method using irradiated PBMCs, IL-2, and IL-15 in a closed system [70]. NK cells were isolated using negative MACS selection and feeder cells were generated by irradiating PBMCs after 3–5 days in culture. Using this approach, the authors achieved an 80- to 200-fold expansion of NK cells that were 75% CD3^−^CD56^+^CD16^+^ and highly cytotoxic against various tumor cell lines. A similar protocol was used by Siegler et al. to achieve single-KIR^+^ NK cells using CliniMACS [71]. After a 268.3-fold expansion with 99% CD3^−^CD56^+^ cells over 19 days using irradiated PBMCs with IL-2, IL-15, and OKT-3, these expanded NK cells were highly cytotoxic against primary human AML blasts both* in vitro* and in an NSG xenograft mouse model. Several other preclinical papers use this approach and demonstrate the ability of PBMCs to act as feeder cells, expanding autologous or allogeneic NK cells [36, 72, 73].

Parkhurst et al. treated eight patients with metastatic melanoma or renal cell carcinoma after lymphodepletion [74]. NK cells were expanded from MACS CD3-depleted PBMCs cultured with irradiated autologous PBMCs, OKT-3, and IL-2 over 21 days. Patients received an average of 4.7 × 10^10^ cells containing 96% CD3^−^CD56^+^ cells. These cells were highly cytotoxic* in vitro* and were deemed safe* in vivo*. However, no clinical responses were observed despite persistence of these cells in the patients.

## 5. “Adherent” Enrichment and Expansion of NK Cells

Some human NK cells have been shown to become “adherent” upon IL-2 activation [75–77]. These adherent NK cells (A-NK) were characterized to possess higher cytotoxicity against a variety of hematopoietic and nonhematopoietic (solid) tumors* in vitro* and in experimental* ex vivo* models than activated nonadherent NK cells (NA-NK) [78–80]. However, these studies did not attempt to enrich and expand the “adherent” enriched NK cells. Here, we report a novel proof-of-principle, cost effective, and simple technology of “adherent” enrichment and subsequent expansion of NK cells for adaptive cell therapy applications utilizing ionomycin and IL-2 priming/activation of NK cells in PBMCs. Costimulatory cell contact-dependent factors as well as a primary mitogenic stimulus are required to jump-start optimal proliferation of resting NK cells [81, 82]. Thus, we used IL-2 to prime the resting NK cells and ionomycin to administer essential costimulatory early signals that bypass the requirement of cell contact-dependent mechanism [83, 84]. Cryopreserved PBMCs from healthy donors and metastatic cancer patients (blood samples from patients were obtained using Thomas Jefferson University Institutional Review Board (IRB) approved protocol) were thawed, washed, counted, and checked for viability (Countess, Life Technologies) and primed with ionomycin (0.75 *μ*g/mL Sigma) and 1000 U/mL IL-2 (Proleukin, Prometheus Laboratories Inc.) in complete SCGM medium (CellGenix) containing 10% Human AB Serum (HABS; Gemini), 1x Glutamax (Gibco by Life Technologies), and 25 U/mL penicillin and 25 mg/mL streptomycin (Cellgro) and cultured in tissue culture-treated flask (Corning) overnight at 37°C incubator with 5% CO_2_. On day 1, floating cells and ionomycin were removed by washing the flask 2-3 times with warm IMDM (Gibco by Life Technologies). The left over adherent cells in the flask were cultured with fresh complete SCGM and 1,000 U/mL IL-2. On days 3 and 5, equal amount of fresh medium was added and the concentration of IL-2 was adjusted to 500 U/mL IL-2. On day 7, adherent enriched and expanded cells were harvested, counted, and checked for percent viability and put back in culture at 1–1.5 × 10^6^ cells/mL in complete SCGM with 500 U/mL IL-2. Every 2-3 days, an equal amount of fresh medium was added and IL-2 concentration was adjusted to 500 U/mL IL-2 throughout the duration of the two-week culture. At the end of the culture period, expanded cells were harvested using HBSS (Corning Cellgro) with 1% HABS and 0.25 M EDTA (Invitrogen by Life Technologies), washed, counted, and checked for viability. In addition, flow cytometry was performed for determining phenotype of cells present in the culture (NK, T, and NK-T cells) using anti-CD3 and CD56 antibodies, expression of CD16 and CD62L, and cytotoxicity against K-562 cells using CFSE/7AAD cytotoxicity assay kit (Abnoa).

**Figure 1:**
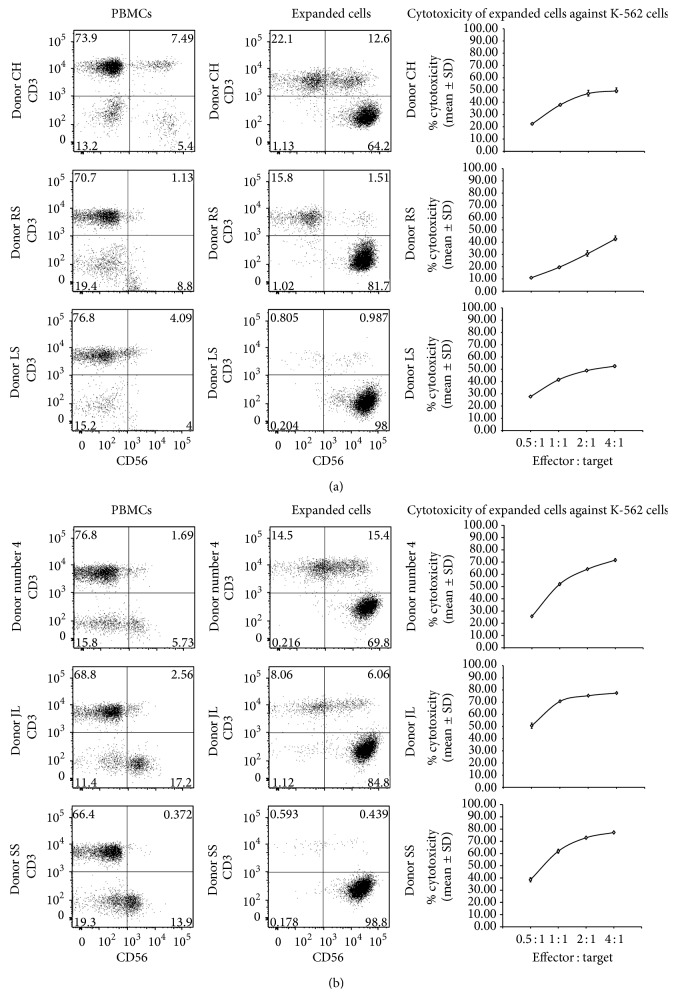
Select (low, medium, and high percent expanded NK cells; Refer to bold cases in Tables 1(a) and 1(b)) flow cytometry dot blots of phenotype of (a) cancer patient and (b) healthy donor PBMCs and corresponding expanded cells, and cytotoxicity of expanded cells against K-562 cells. PBMC populations and two-week expanded cells were stained by incubating with Pacific Blue conjugated anti-CD3 (Clone HIT3a, Biolegend) and Phycoerythrin (PE/Cy7) conjugated anti-CD56 antibodies (Clone MEM-188, Biolegend) and fixed in 2% paraformaldehyde. For all flow cytometry experiments, appropriate IgG isotype controls were used to assess nonspecific staining. Cells were analyzed using a BD LSRII FACS flow cytometer and the data was processed using FlowJo Flow Cytometry Analysis Software (TreeStar Inc.). Cytotoxicity of expanded cells against CFSE-labeled K-562 cells was carried out at various E : T using 7-AAD/CFSE cell-mediated cytotoxicity assay kit (Abnoa).

In three preliminary experiments using our “adherent” enrichment and expansion protocol, NK cells from 25 × 10^6^ PBMCs of each healthy donor, #3, #4, and #21, yielded 68.3% ± 16.5%, 15.8% ± 0.9%, and 89.0% ± 6.2% NK cells with a final expanded NK cell number of 92.5 ± 2.8 × 10^6^, 18.6 ± 0.5 × 10^6^, and 40.0 ± 0.1 × 10^6^, respectively. Based on this, experiments were set out to assess the feasibility of enriching and expanding NK cells from PBMCs of solid metastatic cancer patients. As shown in Table 1(a), nine cancer patients' PBMCs (median 24.6 × 10^6^ cells) were primed overnight. The resulting adherent cells were then enriched and expanded for two weeks, NK cell purity was 82%–98% (median 88%) in eight out of nine cases and 64% in one case (Figure 1(a)). The median percent viability was 92% with median viable total expanded cells of 64 × 10^6^. Within the total cells, the median NK cells were 57 × 10^6^ cells. Based on availability of patient samples, expansions were repeated with PBMCs from patient donors, CH, RS, WN, and MT, and NK cell purity of 67%, 98%, 93%, and 83% was obtained, respectively. Due to enhanced purity of NK cells from cancer patients' PBMCs, healthy donor PBMCs were used to further test and improve the “adherent” NK cells enrichment methodology particularly focusing on the removal of nonadherent cells effectively on day 1 to enhance the purity and number of NK cells (Table 1(b)). In the small-scale attempts, six healthy donors' PBMCs (median 26.2 × 10^6^ cells) were primed overnight. The resulting adherent cells were then enriched and expanded for two weeks. This improved NK cell purity of median 95% was achieved in five out of six cases and it was 70% in one case (Figure 1(b)). The median percent viability was 93% with median viable total expanded cells of 232 × 10^6^. Within the total cells, the median NK cells were 220 × 10^6^ cells. To note, compared with initial preliminary experiments with healthy donor #4 and donor #21, we obtained much greater NK cell purity and yield using the improved washing step on day 1 as shown in the Table 1(b).

Large-scale expansion was also successful using our methodology. Initial 40 million PBMCs of healthy donor SS ^*^ (Table 1(b)) yielded 580 million enriched NK cells with a purity of 99% NK cells after 14 days. Using another healthy donor #21 ^*^, 90 million PBMCs were primed and then “adherent” enriched and expanded; the cell yield was 450 × 10^6^ cells with a purity of 92% NK cells in two weeks (Table 1(b)). To note, in a previous experiment testing of 82 × 10^6^ PBMCs from donor #21 yielded 389 × 10^6^ NK cells with a purity of 69% (with 16% T cells and 17% NK-T cells) and a viability of 94% (data not shown). Notably, as shown in Table 1(b) for the same donor ( ^*^21), the purity and yield of NK cells were greatly enhanced using the improved day 1 washing step, which seems to be critical in obtaining enhanced NK cell purity using the current “adherent” enriched protocol. Importantly, effective removal of floating cells increased not only the purity of NK cells but also the number of NK cells within the expanded total number of cells.

Although the numbers and the composition of adherent cells in the flask on day 1 were not determined in each case, a separate experiment suggests that about 40% of the total NK cells (~10% of total PBMCs) were recovered in the adherent population which expanded to yield the final expanded NK cells. Others reported only a small portion (4–30%) is capable of responding to IL-2 stimulation by adherence [77, 79]. The cell composition of the combined floating and adherent cells was determined by flow cytometry using anti-CD3 and anti-CD56 antibodies after overnight stimulation with IL-2 and ionomycin. It contained 25% NK cells, 43% T cells, 14% NK-T cells, and 18% negative cells. In the floating cells, 15% were NK cells, 57% were NK cells, 16% were NK-T cells, and 13% were negative cells. To note, due to small-scale attempt, we could not obtain sufficient number of adherent cells to carry out the flow cytometry analysis. Based on the above cell composition, we recommend to start with median 220 × 10^6^ “healthy” donor PBMCs in culture to achieve about 2 × 10^9^ NK cells with median purity of 95%, in about two weeks. For “cancer” patients' PBMCs, we recommend median 870 × 10^6^ PBMCs to achieve 2 × 10^9^ NK cells due to the lower starting percentage of NK cells in majority of the patients' PBMCs (Table 1(a)). However, using the improved washing step to remove the nonadherent cells on day 1 as shown in Table 1(b), the starting number of PBMCs can be significantly reduced. These recommendations will be further verified in the future experiments. In addition, it is to note that advantage of our methodology lies on obtaining potent activated A-NK cells which will expectedly reduce the number of required cells to transfer into patients compared to total activated mixed NK cells (adherent and nonadherent NK cells) with potentially reduced potency of NK cells. The expanded cells from both cancer patients and healthy donor PBMCs exhibited cytotoxicity against K-562 cells after expansion for 14 days (Figures 1(a) and 1(b)). In general, the lesser purity of NK cells showed decreased percent killing at various E : T cells tested. In one case, we tested the cytotoxicity of expanded NK cells against allogeneic human uveal melanoma cells and observed potent cytotoxicity (data not shown).

CD16 (Fc*γ* receptor III) is expressed on NK cells involved in facilitating ADCC by binding to the Fc portion of various antibodies and is directly involved in the lysis of some virus-infected cells and tumor cells [85]. Within our enrichment and expansion method, CD16 was widely expressed on NK cells (>80%; data not shown) and was unchanged after* ex vivo* enrichment and expansion of NK cells. However, the change in the MFI of CD16 on expanded NK cells (defined as >30% variation of MFI) from cancer patient donor PBMCs revealed CD16 MFI lower in three, higher in two, and the same in four cases compared to level on NK cells in PBMCs (Table 2). A different pattern was observed in expanded NK cells from healthy donor PBMCs with higher MFI of CD16 in five and the same level in three cases, and none had lower CD16 MFI. Previous studies have shown that NK cell activation by cytokines and target cell stimulation led to marked decreases in CD16, potentially mediated by a disintegrin and metalloproteinase-17 (ADAM-17) upon activation (as well as loss of CD62L; Table 3) [86]. However, we have seen a mixed pattern of expression of CD16 levels, which may be attributed to the culture conditions and also donor variation. In this regard, metalloproteinase inhibitors could be included during* ex vivo* enrichment and expansion of “adherent” NK cells to enhance the level of CD16 [87].

CD62L (L-Selectin) on NK cells plays an important role in its recruitment from the circulation into the bone marrow and secondary lymphoid tissues which after restimulation can accomplish multiple effector tasks [88, 89]. We assessed the change in the expression of CD62L in terms of percent of positive cells and MFI of NK cells within the expanded cells compared to its expression on corresponding NK cells in the PBMCs of healthy donors. Expanded NK cells were of median 30% CD62L^+^ from small-scale enrichment and expansion, expressing percentages consistent with starting NK cells within PBMCs of six donors of median 36% CD62L^+^. After* ex vivo* enrichment and expansion, CD62L^+^ NK cells were higher in two, lower in three, and the same in three cases compared to percent CD62L^+^ NK cells in PBMCs. However, change in the MFI of CD62L on expanded NK cells (defined as >30% variation of MFI after expansion) was lower in five and remained the same in three cases. Considering such reduction of CD62L expression on the expanded adherent NK cells, nicotinamide (NAM) that is shown to substantially increase CD62L expression on NK cells could be tested with these cells [90, 91]. To note, we did not assess the CD62L expression on NK cells of cancer patients, which can be assessed in future experiments.

## 6. Conclusions

As highlighted in Table 4, among the various approaches of NK cell isolation and expansion, tested in the clinic, the use of feeder cells has been most effective in selectively expanding NK cells from healthy donor and patient's PBMCs. More consistent and higher cell numbers have been achieved compared to previous protocols using cytokines alone. However, the addition of feeder cells into a protocol further complicates the translation of these methods. The majority of these protocols use cytokines, and some required CliniMACS isolation methods to achieve optimal NK expansion. The introduction of these variables can result in greatly increased costs and more regulation to comply with when conducting a clinical trial which makes it difficult to move on to multicenter trials. The “adherent” enrichment and expansion of NK cells we describe offer a simple, cost-effective, reproducible, and translatable procedure with a high purity and potency of NK cells compared to other methodologies for personalized adoptive cell therapy. Future work with this method could easily incorporate agents that modify the phenotype of NK cells that could favor homing and enhance multiple facets of effector functions [90–92]. In addition, this method can also be tested in large scale with patient PBMCs for translating into clinical application.

## Figures and Tables

**(a):** 

Donor	PBMCs			Enriched and expanded cells on day 7		Enriched and expanded cells on day 14					
Viable cell count	Percent viability	Percent NK cells (CD3^−^CD56^+^)	Viable cell count	Percent viability	Total viable cell count	Viable NK cell count	Percent NK cells (CD3^−^CD56^+^)	Percent T cells (CD3^+^CD56^−^)	Percent NK-T cells (CD3^+^CD56^+^)	Percent viability

SB	20.0 × 10^6^	82	30.5	14.0 × 10^6^	88	72.0 × 10^6^	63.3 × 10^6^	87.9	9.2	1.5
CH	32.0 × 10^6^	87	5.4	9.9 × 10^6^	78	108.0 × 10^6^	69.3 × 10^6^	**64.2**	**22.1**	**12.6**
TP	10.0 × 10^6^	90	7.9	5.6 × 10^6^	58	36.0 × 10^6^	30.8 × 10^6^	85.5	12.2	1.6
RS	24.0 × 10^6^	84	8.8	12.0 × 10^6^	93	136.0 × 10^6^	111.1 × 10^6^	**81.7**	**15.8**	**1.5**
JB	24.6 × 10^6^	87	14.3	16.8 × 10^6^	84	64.0 × 10^6^	56.6 × 10^6^	88.4	4.1	7.0
WN	25.0 × 10^6^	92	25.4	9.4 × 10^6^	89	70.0 × 10^6^	66.7 × 10^6^	95.3	3.2	1.0
GW	23.5 × 10^6^	94	6.4	12.0 × 10^6^	92	30.0 × 10^6^	27.2 × 10^6^	90.6	6.9	0.5
MT	29.5 × 10^6^	91	14.3	22.0 × 10^6^	82	54.0 × 10^6^	47.0 × 10^6^	87.0	5.5	6.4
LS	25.0 × 10^6^	87	4.0	13.0 × 10^6^	92	40.0 × 10^6^	39.2 × 10^6^	**98.0**	**0.8**	**1.0**

**(b):** 

Donor	PBMCs			Enriched and expanded cells on day 7		Enriched and expanded cells on day 14					
Viable cell count	Percent viability	Percent NK cells (CD3^−^CD56^+^)	Viable cell count	Percent viability	Total viable cell count	Viable NK cell count	Percent NK cells (CD3^−^CD56^+^)	Percent T cells (CD3^+^CD56^−^)	Percent NK-T cells (CD3^+^CD56^+^)	Percent viability

2	26.4 × 10^6^	80	13.1	42.0 × 10^6^	79	216.0 × 10^6^	203.5 × 10^6^	94.2	4.2	0.8
JL	27.0 × 10^6^	84	17.2	19.0 × 10^6^	94	180.0 × 10^6^	152.6 × 10^6^	**84.8**	**8.1**	**6.1**
4	28.0 × 10^6^	77	5.7	50.0 × 10^6^	95	409.2 × 10^6^	285.6 × 10^6^	**69.8**	**14.5**	**15.4**
AS	20.0 × 10^6^	79	17.0	17.4 × 10^6^	93	320.0 × 10^6^	304.6 × 10^6^	95.2	1.3	2.8
SS	25.0 × 10^6^	86	15.7	21.0 × 10^6^	93	201.6 × 10^6^	197.4 × 10^6^	97.9	0.8	0.5
^*^SS	40.0 × 10^6^	85	13.9	48.0 × 10^6^	95	580.0 × 10^6^	573.0 × 10^6^	**98.8**	**0.6**	**0.4**
21	26.0 × 10^6^	70	19.8	19.2 × 10^6^	85	248.0 × 10^6^	235.4 × 10^6^	94.9	2.5	2.4
^*^21	96.0 × 10^6^	91	19.8	75.0 × 10^6^	90	451.1 × 10^6^	415.0 × 10^6^	92.0	2.6	4.7

^*^Large-scale expansion.

Bold numbers represent low, medium and high percent expanded NK cells with respective presence of T cells and NK-T cells.

**Table 2:** Change in the MFI of CD16 on CD3^−^CD56^+^ cells of PBMCs and “adherent” enriched and expanded cells. Analysis of CD16 expression on CD3^−^CD56^+^ cells of PBMCs and “adherent” enriched and expanded cells. PBMC populations and two-week expanded cells were stained with Pacific Blue conjugated anti-CD3 (Clone HIT3a, Biolegend), Phycoerythrin-Cyanine 7 (PE/Cy7) conjugated anti-CD56 (Clone MEM-188, Biolegend), and FITC conjugated anti-CD16 (Clone 3G8, Biolegend) antibodies and fixed in 2% paraformaldehyde. For all flow cytometry experiments, appropriate IgG isotype controls were used to assess nonspecific staining. Cells were analyzed using a BD LSRII flow cytometer (BD Biosciences) and the data was analyzed using FlowJo Flow Cytometry Analysis Software (TreeStar Inc.).

Donor	Percent NK cells in PBMCs	Percent NK cell purity on day 14	CD16 MFI of NK cells in PBMCs	CD16 MFI of day 14 NK cells	Change in CD16^+^ NK cell MFI after enrichment and expansion for 14 days
Cancer patient donor PBMCs					
SB	30.5	87.9	22,746	11,512	49.4% lower
CH	5.4	64.2	13,600	24,478	80.0% higher
TP	7.9	85.5	10,198	10,833	6.2% higher
RS	8.8	81.7	15,150	10,545	30.4% lower
JB	14.3	88.4	10,644	8,191	23.0% lower
WN	25.4	95.3	11,369	19,061	67.7% higher
GW	6.4	90.6	14,215	3,221	77.3% lower
MT	14.3	87.0	9,624	12,104	25.8% higher
LS	4.0	98.0	10,698	10,833	1.2% higher

Healthy donor PBMCs					
2	13.1	94.2	25,808	36,272	40.5% higher
JL	17.2	84.8	14,687	19,563	33.2% higher
4	5.7	69.8	13,828	22,281	61.1% higher
AS	17.0	95.2	24,726	22,696	8.2% lower
SS	15.7	97.9	14,761	21,258	44.0% higher
^*^SS	13.9	98.8	14,613	20,062	37.3% higher
21	19.8	94.9	11,484	14,322	24.7% higher
^*^21	19.8	92.0	11,484	10,732	6.5% lower

^*^Large-scale expansion.

**Table 3:** Change in the percent and MFI of CD62L on CD3^−^CD56^+^ cells from healthy donor PBMCs after enrichment and expansion. Analysis of CD62L expression on CD3^−^CD56^+^ cells before and after enrichment and expansion. PBMC populations and two-week expanded cells were stained with Pacific Blue conjugated anti-CD3 (Clone HIT3a, Biolegend), Phycoerythrin-Cyanine 7 (PE/Cy7) conjugated anti-CD56 (Clone MEM-188, Biolegend), and Allophycocyanin (APC) conjugated anti-CD62L (Clone P3.6.2.8.1, eBioscience) antibodies and fixed in 2% paraformaldehyde. For all flow cytometry experiments, appropriate IgG isotype controls were used to assess nonspecific staining. Cells were analyzed using a BD LSRII flow cytometer (BD Biosciences) and the data was analyzed using FlowJo Flow Cytometry Analysis Software (TreeStar Inc.).

Donor	In PBMCs			In expanded NK cells on day 14			Culture-induced change	
	Percent NK cells	Percent CD62L^+^ NK cells	CD62L MFI of NK cells	Percent NK cell purity	Percent CD62L^+^ NK cells	MFI of CD62L	Change in CD62L^+^ NK cells after expansion	Change in CD62L^+^ NK cell MFI after expansion
2	13.1	30.8	5,647	94.2	24.8	4,383	19.5% lower	22.4% lower
JL	17.2	17.3	5,689	84.8	35.3	4,394	104.0% higher	32.8% lower
4	5.7	14.4	2,846	69.8	25.7	3,579	78.5% higher	25.8% higher
AS	17.0	68.5	13,316	95.2	62.9	9,119	8.2% lower	31.5% lower
SS	15.7	69.0	6,672	97.9	34.9	3,373	49.4% lower	49.4% lower
^*^SS	13.9	65.7	6,927	98.8	50.1	5,891	23.7% lower	15.0% lower
21	19.8	36.0	5,891	94.9	14.7	2,953	59.2% lower	49.1% lower
^*^21	19.8	36.0	5,891	92.0	23.6	2,537	34.4% lower	56.9% lower

^*^Large-scale expansion.

**Table 4:** Advantages and disadvantages of methods of NK cell isolation and expansion.

NK cell isolation approach			NK cell expansion approach		
Method	Advantages	Disadvantages	Method	Advantages	Disadvantages
CliniMACS	(i) Allows a highly purified NK cell product (ii) Clinical-grade separation method approved for GMP use	(i) Complicated protocol and increased costs (ii) Two selections may be necessary (CD3 depletion/CD56 positive selection) (iii) Positive selection by anti-CD56 antibody binding could affect NK cell activation	No or brief stimulation	(i) Does not require long-term cell culture (ii) Minimal costs of cytokines and reagents (iii) Individual clinical trials have been able to treat a large number of patients due to minimal labor	(i) Require large number of starting PBMCs (ii) Low percentage and cell number of NK cells (iii) Minimal reactivation of NK cells that may be preventing antitumor response

OKT-3	(i) Widely used antibody to eliminate CD3^+^ cells from culture (ii) GMP-grade antibody is available (iii) Eliminates need for expensive MACS procedure and simplifies the process	(i) Percent of NK cells in final culture varies (ii) Antibody needed for several days, while T cells are present and they could compete with NK cells for cytokines, limiting their effect	Cytokines	(i) GMP-grade IL-2 is widely available and affordable, allowing effective translation (ii) Capable of generating highly activated NK cells from patients (iii) Large-scale expansions have been successful and reproducible	(i) Purity of final NK cell product has traditionally been suboptimal (ii) Low final NK cell number compared to feeder cell expansion (iii) Cytokines other than IL-2 are more expensive and most are not GMP-grade yet.

Adherent selection	(i) Minimal cost needed (ii) Simple and reproducible (iii) Overnight selection, allowing quick elimination of T cells and preventing T cells from taking up cytokines/media (iv) Upon enrichment IL-2 is used for expansion without any additional feeder cells	(i) Adherent culture flasks are needed instead of culture bags (ii) Large-scale enrichment and expansion need to be tested using patient PBMCs reproducing similar NK cell purity with healthy donor PBMCs	Feeder cells	(i) Highest number of final NK cells has been achieved using this approach (ii) Ability to present various activating molecules to NK cells by genetically modifying feeder cells (iii) Some protocols require no further isolation	(i) GMP-grade feeder cells must be developed and maintained (ii) Most protocols also require IL-2 (iii) Final product must be ensured to be deficient of all feeder cells before returning to the patient

*Note.* The table above summarizes the pros and cons of choosing a particular NK cell expansion method in NK cell isolation and NK cell expansion. Considerations for this table include NK cell purity of final product, NK cell viability, reproducibility, cost-effectiveness, GMP protocols, and clinical application.
